# The L-Type Amino Acid Transporter LAT1—An Emerging Target in Cancer

**DOI:** 10.3390/ijms20102428

**Published:** 2019-05-16

**Authors:** Pascal Häfliger, Roch-Philippe Charles

**Affiliations:** Institute of Biochemistry and Molecular Medicine, and Swiss National Center of Competence in Research (NCCR) TransCure, University of Bern, Bühlstrasse 28, CH-3012 Bern, Switzerland; ph2548@cumc.columbia.edu

**Keywords:** l-type amino acid transporter LAT1, cancer, preclinical studies, targeted therapy, inhibitor

## Abstract

Chronic proliferation is a major hallmark of tumor cells. Rapidly proliferating cancer cells are highly dependent on nutrients in order to duplicate their cell mass during each cell division. In particular, essential amino acids are indispensable for proliferating cancer cells. Their uptake across the cell membrane is tightly controlled by membrane transporters. Among those, the L-type amino acid transporter LAT1 (*SLC7A5*) has been repeatedly found overexpressed in a vast variety of cancers. In this review, we summarize the most recent advances in our understanding of the role of LAT1 in cancer and highlight preclinical studies and drug developments underlying the potential of LAT1 as therapeutic target.

## 1. Introduction: LAT1 Transporter

Sustained proliferative signaling is a major hallmark of cancer [1]. These signals initiate tumor cells division, resulting in rapid growth in an uncontrolled way. The duplication of cell mass is highly demanding of energy and depends on the availability of nutrients in the tumor microenvironment. Among these nutrients, glucose is the most famous leading to the use of glucose uptake imaging to evidence tumors and metastases. Apart from glucose, essential amino acids (EAA) were described to be indispensable for cell proliferation in vitro by Harry Eagle in 1955, since HeLa cells rapidly died when a single EAA was omitted from the medium [2]. Subsequently, EAA uptake was demonstrated to be higher in tumors in vivo compared to normal tissues [3,4,5], suggesting that increased EAA uptake is required to maintain protein synthesis in highly proliferative cancers [6]. EAA uptake across the plasma membrane is tightly controlled by amino acid transporters or Solute Carriers (SLC). The L-type amino acid transporter family serves as an important route for EAA entry into cells and consists of four members (LAT1–4). In a previous extensive review, Wang and Holst reported the literature demonstrating that among the four LAT transporters, LAT1 was predominantly found to be overexpressed in a variety of cancers [7]. LAT1 (*SLC7A5*) contains 12 transmembrane domains and covalently binds to 4F2hc (*SLC3A2*), which incorporates LAT1 into the plasma membrane resulting in its functional expression [8]. LAT1 mediates the influx of neutral EAA (leucine, isoleucine, phenylalanine, methionine, histidine, tryptophan, valine, and tyrosine) into cells [9,10] in exchange for the efflux of intracellular substrates (EAA or glutamine) [11,12], thus acting as an antiporter. This review will give an update on the role of LAT1 in cancer and its potential as a therapeutic target or drug carrier in different malignancies.

## 2. LAT1 Expression in Cancer

Studies that demonstrated an increased LAT1 expression comparing late-stage vs. early-stage diseases or between malignant vs. normal tissues are listed in Table 1. *SLC7A5/*LAT1 was found overexpressed in a wide range of solid tumors including the most frequently diagnosed cancers such as breast cancer [13,14], prostate cancer [15,16,17], and lung cancer [18,19,20,21,22,23,24,25,26,27,28,29,30]. Additionally, LAT1 was found overexpressed in bladder cancer [31], bone cancer [32], brain tumors [33,34], gynecological cancers [35,36], esophagus cancers [37,38,39], gastrointestinal cancers [40,41], liver [42,43,44,45], pancreas [46,47], head and neck cancers [48], kidney cancer [49], leukemia [50], skin cancer [51,52,53,54], myeloma [55,56], sarcoma [32], thymic carcinoma [57], and thyroid cancer [58,59]. Most of these studies detected LAT1 at the protein level in patient-derived tissue microarrays and benign tissue was used as the control. Importantly, LAT1 was not detected by immunohistochemical staining in most non-cancerous tissues, however, few studies reported a positive staining in activated lymphocytes [60], uterine glands [36], as well as in the basal layer of the esophageal and uterine mucosa [35,37,39]. In several studies LAT1 expression was not significantly associated with tumor stage, which was described in biliary tract cancer [61], ovarian cancer [62], lung cancer [63], and mesothelioma [64]. Therefore, since LAT1 expression does not uniformly correlate with increased tumor stages, its functional consequence needs to be evaluated for each cancer in preclinical models.

The majority of the studies are correlative and did not validate the tumorigenic role of LAT1. However, few studies are particularly comprehensive because they provide additional functional validation of LAT1 in cancer cell lines (breast [14], endometrial [36], leukemia [50], lung [30], melanoma [52,53], and thyroid [59] cancer). The functional validation of LAT1 in each of these studies (pharmacological inhibition or downregulation by RNA interference) will be discussed in this review in Section 3 and Section 4. 

The mechanism(s) of LAT1 upregulation in these studies is not well understood. One hypothesis was put forward: hypoxia could be involved since it was able to increase *SLC7A5* transcription in vivo through transcriptional activation by HIF2α [98]. Interestingly, LAT1 expression was significantly correlated with VEGF expression in several publications [19,25,46,57] and VEGF is a well-described hypoxia marker [99,100], which indicates that hypoxia might indeed be partially responsible for the upregulation of LAT1. Low nutrient levels in tumors might elevate LAT1 levels as well, because leucine or glutamine starvation was shown to increase LAT1 expression in vitro [93,101]. Gene amplification might play a minor role in LAT1 overexpression since these events, to our knowledge, have not been described so far. However, *MYC* amplification, that frequently occurs in cancers, is a potential genetic alteration leading to LAT1 overexpression since c-MYC was shown to enhance LAT1 promoter activity in vitro [102]. Also, the use of a MEK1/2 inhibitor significantly reduced *Slc7a5* transcription in mouse thyroid tumors model [59] emphasizing the role of the RAS-MEK-ERK pathway in LAT1 regulation. This underscores the fact that LAT1 elevated expression is a frequent event observed during cancer transformation.

High LAT1 expression was associated with a significantly shorter survival in the majority of cancers in which LAT1 was upregulated (summarized in Table 1). These studies cover the most frequently diagnosed cancers such as breast cancer [13,72], prostate cancer [16,17], colorectal cancer [77], and lung cancer [18,19,20,21,23,25,27,28,29,30,63,87]. Importantly, in several of these studies a positive correlation between LAT1 levels and Ki67-positive cells was described [13,29,41,56,62], suggesting that LAT1 might support growth of highly proliferating tumors. In contrast, few studies reported no significant association between LAT1 expression and patient survival, which was described in studies conducted in lung cancer [22,26] and cutaneous angiosarcoma [103]. However, the study about cutaneous angiosarcoma might be biased because the sample size (*n* = 52) was relatively small in order to be fully representative [103]. Overall, LAT1 has been proposed as a promising prognostic biomarker to predict the outcome in a variety of different cancer types, with the exception of lung cancers, in which some discrepancies among different publications exist. Therefore, future studies are required in lung cancer to assess whether LAT1 expression alone can serve as prognostic biomarker or whether additional markers in combination with LAT1 need to be considered. 

## 3. Downregulation of LAT1 and Tumor Cell Growth

In order to address whether a causal relationship between LAT1 and tumor growth exists, LAT1 expression was reduced by gene downregulation in multiple studies. LAT1 downregulation by RNA interference was shown to impair growth of breast [14], endometrial [36], gastric [83], oral [84], ovarian [90], pancreatic [92], and prostate [17,93,94] cancer cell lines. The studies conducted in breast [14] and endometrial [36] cancer cell lines are particularly informative because they additionally demonstrate an upregulation of LAT1 in patient-derived tumor tissues, further suggesting that LAT1 plays a functional role in these cancers. In line with these results, zinc finger nuclease-mediated knockout of LAT1 in lung and colorectal cancer cell lines significantly reduced cell proliferation [78]. Moreover, LAT1 downregulation impaired migration and invasion of gastric and prostate cancer cell lines [17,83], suggesting that the increased LAT1 expression detected in metastatic lesions compared to the primary site [104] might play a role in the formation of metastases. 

## 4. Drug-Mediated Inhibition of LAT1

Based on the numerous studies demonstrating that LAT1 is overexpressed in a variety of cancers, and the efficacy of downregulating LAT1 to obtain tumor cell growth reduction, efforts were undertaken in order to synthesize and characterize potent inhibitors of LAT1-mediated amino-acids transport (summarized in Table 2). Among them, BCH (2-aminobicyclo[2.2.1]heptane-2-carboxylic acid) has been shown to reduce growth of a variety of different cancer cells including breast [14,73], prostate [93,95], and lung [30] cancer cell lines among others (see Table 2). However, BCH is a rather unspecific L-type amino acid transporter inhibitor that blocks LAT1–4 [9,105,106,107]. Thus, it remains unclear in these studies whether the inhibition of LAT1 alone is sufficient to affect cell proliferation. In 2010, Oda et al. published a compound (KYT-0353 or JPH203) that selectively inhibited LAT1 with an IC_50_ value of 0.06 μM in HT-29 colon cancer cells that did not block LAT2 at this concentration [79]. Importantly, JPH203 inhibited HT-29 colon cancer cell proliferation with an IC_50_ of 4.1 μM and significantly reduced tumor growth in vivo in a xenograft model [79]. JPH203 is a tyrosine analogue that was designed based on the structure of the thyroid hormone triiodothyronine (T3), which is a substrate of LAT1 and LAT2 [108,109]. JPH203 was subsequently tested in other cancer types and successfully reduced growth of brain [71], gastric [80], head and neck [86], leukemia [50], lung [78], kidney [78], prostate [95], thymic carcinoma [96], and thyroid cancer [59] cell lines. Importantly, LAT1 inhibition by JPH203 has demonstrated a convincing potential in vivo as shown by a diminished tumor growth in xenograft models of human leukemia cells [50] and colon cancer cells [79]. Moreover, we have recently shown that JPH203 induced a cytostatic growth arrest in a genetically engineered mouse model (GEMM) of anaplastic thyroid carcinoma [59] where the immune system of the mice remains intact. Interestingly, LAT1 expression level might not be the only factor that determines a therapeutic response to JPH203 since we observed that LAT1 expression levels do not always predict JPH203 sensitivity in thyroid cancer cells [59]. Similar results were independently obtained recently in gastric cancer cells by another group [80]. Additionally, we observed in our study that the effect of JPH203 in vitro is highly dependent on the concentration of LAT1 substrates in the culture medium, which suggests that JPH203 acts as a competitive inhibitor of LAT1 [59]. Importantly, most conventional culture media contain EAA concentrations that greatly exceed the physiological levels found in plasma. Therefore, studies that address the effect of JPH203 in vitro should be performed in customized medium that mimics more closely the clinical conditions. It is crucial to mention that plasma levels of LAT1 substrate amino acids are significantly changed in cancer patients: plasma LAT1 substrates were found increased in lung [110,111], prostate [112], and breast cancer patients [113,114]. Furthermore, in breast cancer EAA were significantly increased in the most aggressive tumor subtype compared to the least aggressive subtype [113]. In contrast, LAT1 substrates were found decreased in gastrointestinal [115,116], myeloma [117], as well as pancreatic cancer patients [118]. The reason(s) for the cancer-specific alterations of plasma EAA remains largely unknown, since the regulation of plasma amino acid concentrations in cancer patients is highly complex. There are multiple factors such as diet, whole-body protein metabolism, and amino acid consumption of the tumor that influence the plasma EAA level. As an example, late-stage cancer patients are often malnourished due to lack of appetite, which can result in decreased plasma levels of LAT1 substrates [119,120]. It is currently poorly understood whether alterations of plasma LAT1 substrates are consistent in the tumor microenvironment and whether plasma EAA can predict the response to LAT1 inhibition. Since JPH203 acts as a competitive inhibitor, there is a possibility that high intratumoral LAT1 substrate amino acids may reduce or completely overcome the effect of the drug. Therefore, future studies are required in order to address whether the intratumoral concentration of LAT1 substrates influences the efficacy of competitive LAT1 inhibitors such as JPH203 in vivo. Additionally, to our knowledge, the effect of plasma EAA on outcome of patients who are not malnourished has not been investigated so far. Therefore, future studies should address the effect of plasma EAA levels on outcome of patients and whether plasma EAA can be used as predictor for response to LAT1 inhibitors, particularly in the case of the competitive LAT1 inhibitor JPH203.

Kongpracha et al. synthesized another LAT1-specific inhibitor (SKN103) based on the structure of T3 and demonstrated that this compound inhibited the proliferation of pancreatic cancer cells as well as squamous cell carcinoma cells. Furthermore, the authors showed that SKN103 in combination with cisplatin additively reduced cell growth [88] suggesting that SKN103 could enhance the therapeutic efficacy of chemotherapies in patients. SKN103 has not been tested in vivo so far.

Singh et al. reported recently the discovery of novel LAT1 inhibitors by using a structure-based approach [121]. Unlike JPH203, which was rationally designed based on the structure of tyrosine, Singh et al. used an unbiased approach by performing docking-based virtual screening of commercially available libraries. By using this elegant method, the authors identified 13 previously unknown LAT1 ligands. Dose-response curves uncovered two novel highly potent LAT1 inhibitors with IC_50_ values of 0.64 μM and 1.48 μM [121]. It will be very interesting to test the anticancer properties of these promising candidates in future studies. Moreover, the structure of these two compounds will open avenues for the rational design of novel LAT1 inhibitors, which underscores the importance of this study. 

Napolitano and colleagues aimed at finding novel LAT1 inhibitors with prolonged activity by blocking LAT1 irreversibly. To reach this goal, they screened compounds based on dithiazole and dithiazine scaffold, which have the potential to form disulfide bridges with C407, which is a key residue of the substrate binding site [123]. Interestingly, out of 59 compounds they identified eight compounds that almost completely (>90%) inhibited LAT1 transport activity at 100 μM. Furthermore, dose-response curves revealed that the most potent inhibitors exhibited IC_50_ values of 0.98 μM and 0.89 μM [76]. Both compounds induced cell death of cervical cancer cells (SiHa) at a 10 μM concentration and this effect was still observed after washing out the compounds [76], as expected due to the covalent binding to LAT1 by these compounds. The breakthroughs in the discovery of highly potent covalent inhibitors of LAT1 will enable the design of novel drugs. It will be very interesting in the future to further evaluate all of these compounds in preclinical studies in order to test their anticancer effects, particularly in comparison with the currently available competitive inhibitors. Covalent inhibitors of LAT1 have the great advantage that their potency is not influenced by the availability of other LAT1 substrates, which is not the case for competitive inhibitors. Therefore, covalent inhibitors might demonstrate a higher efficacy in preclinical studies compared to the currently available competitive inhibitors. 

Recently, Ueda et al. tested whether targeting LAT1 by monoclonal anti-LAT1 antibodies might be a potential therapeutic approach. The authors found that the anti-LAT1 antibodies reduced proliferation of gastric and lung cancer cells in vitro and growth of colon cancer cells in vivo [81], suggesting that targeting LAT1 by monoclonal antibodies might be an alternative therapeutic option. 

Taken together, results of the current preclinical studies assessing inhibition of LAT1 are promising and suggest that targeting LAT1 might be a well-tolerated effective strategy in different cancer types.

## 5. LAT1 Binding Nanoparticles

Several studies have been published in the past recent years addressing the potential of LAT1 for enhanced delivery of nanoparticles into cancer cells. In two independent studies, nanoparticles were conjugated with glutamate as ligand for LAT1: Li et al. showed that glutamate-conjugated paclitaxel nanoparticles (SPG25 NPs) exhibited increased antitumor efficiency compared to unconjugated nanoparticles in breast cancer cells in vitro and in vivo [74]. Similarly, glutamate-conjugated docetaxel-loaded liposomes manifested an increased cytotoxicity in vitro and higher accumulation in the brain compared to unconjugated liposomes [69]. Other investigators have successfully used dopamine-conjugated nanoparticles to enhance the uptake in LAT1 overexpressing cancer cells: Ong et al. found that conjugation of L-dopa to anisotropic gold nanoparticles (AuNPs) mediated selective photothermal ablation of breast cancer cells and sensitized cells to chemotherapy [75]. Additionally, L-dopa-conjugated liposomes demonstrated increased efficacy both in vitro as well as in vivo compared to non-targeting liposomes in glioblastoma cells [70]. Taken together, these recent studies suggest that LAT1-targeting nanoparticles might be a promising approach to increase the efficacy of nanoparticle-delivered drugs. This approach might be particularly interesting for treatments that are associated with severe side effects, since the enhanced accumulation of LAT1-targeting nanoparticles in cancer cells might reduce accumulation in non-cancerous tissues. Further studies, particularly in preclinical models, are warranted to confirm the increased efficacy of LAT1-targeting nanoparticles. 

## 6. LAT1 Inhibition in Clinical Studies

The competitive LAT1 inhibitor JPH203 was recently evaluated in a first-in-human Phase 1 clinical trial: 17 patients diagnosed with advanced solid tumors were enrolled and partial response or stable disease was achieved in six patients. Common treatment-related adverse events included malaise, nausea, and a grade 1 or 2 fever. Four out of the six responders were diagnosed with biliary tract cancer, in which plasma levels of LAT1 substrates remained high [122]. Therefore, the authors concluded that high plasma LAT1 substrate amino acids could be an important factor that determines the efficacy of JPH203 in biliary tract cancer. However, since only 17 patients were enrolled in this phase 1 clinical trial, these findings need to be validated in a larger cohort of patients. Importantly, based on the promising results of the Phase 1 clinical trial, JPH203 is currently being evaluated in a Phase 2 clinical trial in patients with advanced biliary tract cancers (UMIN Clinical Trials Registry UMIN000034080). Future studies that identify predictive biomarkers for response to LAT1 inhibitors in patients are highly warranted. To our knowledge, JPH203 is currently the only LAT1 inhibitor that is being evaluated in clinical trials. 

## 7. Conclusions

The tumor-specific overexpression of LAT1 has been demonstrated in a variety of different cancer types and is often associated with a worse prognosis. Due to the fact that LAT1 upregulation seems to be a general phenomenon in cancer, targeting LAT1 might be a promising anticancer strategy. As a result, extensive research is currently underway in order to develop potent and specific LAT1 inhibitors. Recently, great efforts by many groups worldwide have led to the discovery of highly potent and selective LAT1 inhibitors that exerted promising anticancer effects in preclinical as well as clinical studies. Of particular interest are the promising results observed with JPH203 in a recent Phase 1 clinical trial, in which JPH203 was effective against biliary tract cancer. Covalent inhibitors might exert an enhanced anticancer efficacy compared to the currently available competitive inhibitors, since covalent inhibitors irreversibly block the transporter. Furthermore, several research groups evidenced that LAT1-targeting nanoparticles are highly effective for LAT1-overexpressing tumors.

Taken together, currently available LAT1 inhibitors as well as LAT1-targeting nanoparticles have shown great success in different preclinical and clinical studies.

The challenge of future studies will be to predict which patients will benefit most from LAT1 inhibitors. This is particularly important for competitive LAT1 inhibitors, since it is currently unknown whether the levels of LAT1 substrates in the tumor microenvironment influence the efficacy of the treatment.

## Figures and Tables

**Table 1:** Role of l-type amino acid transporter (LAT1) in different tumor types.

Cancer Type	Overexpression	Prognosis	Downregulation	Inhibition
Biliary tract	[65]	[60,61,65]		[61] (BCH)
Bladder	[31]			[66] (BCH)
Bone	[32]			
Brain	[33,34]	[34]		[67,68] (BCH) [69,70] (NP) [71] (JPH)
Breast	[13,14]	[13,72]	[14]	[14,73] (BCH), [74,75] (NP)
Cervical	[35]			[76] (compound5)
Colorectal		[77]	[78] (KO)	[78,79,80] (JPH), [81] (Ab)
Endometrial	[36]	[36]	[36]	[36] (BCH)
Esophageal	[37,38,39]	[38]		[82] (BCH)
Gastric	[40,41]	[41]	[83]	[81] (Ab), [80] (JPH)
Head and neck	[48]	[48]	[84]	[67,85] (BCH), [86] (JPH)
Kidney	[49]	[49]		[78] (JPH)
Leukemia	[50]			[50] (JPH)
Liver	[42,43,44,45]	[43,45,65]		
Lung	[18,19,20,21,22,23,24,25,26,27,28,29,30]	[18,19,20,21,23,25,27,28,29,30,63,87]	[78] (KO)	[30] (BCH), [78] (JPH), [88] (SKN103), [81] (Ab)
Melanoma	[51,52,53]	[51]		[52,53] (BCH)
Mesothelioma		[64]		
Myeloma	[55,56]	[56]		
Ovarian		[62,89]	[90]	[91] (BCH)
Pancreatic	[46,47]	[46,47,92]	[92]	[88] (SKN103)
Prostate	[15,16,17]	[16,17]	[17,93,94]	[95] (JPH, BCH), [93] (BCH)
Sarcoma	[32]			[67] (BCH)
Skin	[54]			
Thymic carcinoma	[57]			[96] (JPH)
Thyroid	[58,59]	[59]		[59] (JPH)
Urinary tract	[97]	[97]		

Overexpression: references indicate LAT1 upregulation during cancer progression (late-stage vs. early-stage or cancer vs. normal tissue); Prognosis: references indicate shorter survival of patients with high LAT1 expression in tumors; Downregulation: references indicate that LAT1 downregulation inhibited cancer cell growth (KO = LAT1 knockout cell line); Inhibition: references indicate that pharmacological inhibition of LAT1 by either the unspecific LAT inhibitor BCH (2-aminobicyclo[2.2.1]heptane-2-carboxylic acid) or the LAT1 specific inhibitor JPH203 or nanoparticles (NP) or indicated compound reduced cell growth.

**Table 2:** Current LAT1-targeting therapies.

Class	Compound	Mechanism of Action	In Vitro: Proliferation Inhibition	Preclinical/Clinical Studies
non-selective LAT1 substrate	BCH	Competes with the uptake of LAT1 substrates by binding to LAT1 and being transported into the cells	biliary tract [61] bladder [66] brain [67,68] breast [14,73] endometrial [36] esophageal [82] head and neck [67,85] lung [30] melanoma [52,53] ovarian [91] prostate [93,95] sarcoma [67]	preclinical: biliary tract [61] esophageal [82]
LAT1 inhibitors	compounds **28**/**36**/**42** [121]	Binds to LAT1 thereby impairing transport of LAT1 substrates		
	SKN103	Non-transportable blocker that inhibits LAT1 in a competitive manner	pancreatic/squamous cell carcinoma [88]	
	JPH203	Non-transportable blocker that inhibits LAT1 in a competitive manner	brain [71] colorectal [78,79,80] gastric [80] head and neck [86] kidney [78] leukemia [50] lung [78] prostate [95] thymic carcinoma [96] thyroid [59]	preclinical: colorectal [79] leukemia [50] thyroid [59] clinical phase 1: [122] solid tumors
	compounds 5/17	Binds covalently to the substrate binding site of LAT1 (residue C407) thereby impairing transport of LAT1 substrates	cervical [76]	
mAb against LAT1	Ab1	Binds to LAT1 thereby impairing transport of LAT1 substrates	gastric, lung [81]	preclinical: colorectal [81]
nanoparticles	SPG25 NPs	↑ accumulation in LAT1-expressing cells	breast [74]	preclinical: breast [74]
	AuNU	↑ accumulation in LAT1-expressing cells	breast [75]	
	Amphi-DOPA	↑ accumulation in LAT1-expressing cells	glioblastoma [70]	glioblastoma [70]
	DTX-TGL	↑ accumulation in LAT1-expressing cells	glioma [69]	
